# New Cationic *fac*-[Re(CO)_3_(deeb)B2]^+^ Complex, Where B2 Is a Benzimidazole Derivative, as a Potential New Luminescent Dye for Proteins Separated by SDS-PAGE

**DOI:** 10.3389/fchem.2021.647816

**Published:** 2021-03-25

**Authors:** Alexander Carreño, Manuel Gacitúa, Eduardo Solis-Céspedes, Dayán Páez-Hernández, Wesley B. Swords, Gerald J. Meyer, Marcelo D. Preite, Ivonne Chávez, Andrés Vega, Juan A. Fuentes

**Affiliations:** ^1^ Center of Applied NanoSciences (CANS), Facultad de Ciencias Exactas, Universidad Andres Bello, Santiago, Chile; ^2^ Facultad de Química y Biología, USACH, Santiago, Chile; ^3^ Escuela de Bioingeniería Médica, Facultad de Medicina, Universidad Católica del Maule, Talca, Chile; ^4^ Laboratorio de Bioinformática y Química Computacional, Facultad de Medicina, Universidad Católica del Maule, Talca, Chile; ^5^ Department of Chemistry, University of North Carolina at Chapel Hill, Chapel Hill, NC, United States; ^6^ Departamento de Química Orgánica, Facultad de Química y Química y de Farmacia, Pontificia Universidad Católica de Chile, Santiago, Chile; ^7^ Departamento de Química Inorgánica, Facultad de Química y Química y de Farmacia, Pontificia Universidad Católica de Chile, Santiago, Chile; ^8^ Departamento de Ciencias Químicas, Facultad de Ciencias Exactas, Universidad Andres Bello, Viña del Mar, Chile; ^9^ Centro para el Desarrollo de la Nanociencia y la Nanotecnología Cedenna, Santiago, Chile; ^10^ Laboratorio de Genética y Patogénesis Bacteriana, Facultad de Ciencias de la Vida, Universidad Andrés Bello, Santiago, Chile

**Keywords:** rhenium(I) tricarbonyl, cyclic voltammetry, relativistic DFT, ZFS, protein dye, SDS-PAGE, protein, fluorescent stain

## Abstract

Sodium-dodecyl-sulfate polyacrylamide gel electrophoresis (SDS-PAGE) can be used to separate proteins based mainly on their size such as in denaturing gels. Different staining methods have been reported to observe proteins in the gel matrix, where the most used dyes are generally anionic. Anionic dyes allow for interactions with protonated amino acids, retaining the dye in the proteins. Fluorescent staining is an alternative technique considered to be sensitive, safe, and versatile. Some anionic complexes based on d^6^ transition metals have been used for this purpose, where cationic dyes have been less explored in this context. In this work, we synthesized and characterized a new monocationic rhenium complex *fac*-[Re(CO)_3_(**deeb**)**B2**]^+^ (where **deeb** is 4,4′-bis(ethoxycarbonyl)-2,2′-bpy and **B2** is 2,4-di-*tert*-*butyl*-6-(3H-imidazo[4,5-c]pyridine-2-yl)phenol). We carried out a structural characterization of this complex by MS^+^, FTIR, ^1^H NMR, D_2_O exchange, and HHCOSY. Moreover, we carried out UV-Vis, luminescence, and cyclic voltammetry experiments to understand the effect of ligands on the complex’s electronic structure. We also performed relativistic theoretical calculations using the B3LYP/TZ2P level of theory and R-TDDFT within a dielectric continuum model (COSMO) to better understand electronic transitions and optical properties. We finally assessed the potential of *fac*-[Re(CO)_3_(**deeb**)**B2**]^+^ (as well as the precursor *fac*-Re(CO)_3_(**deeb**)Br and the free ligand **B2**) to stain proteins separated by SDS-PAGE. We found that only *fac*-[Re(CO)_3_(**deeb**)**B2**]^+^ proved viable to be directly used as a luminescent dye for proteins, presumably due to its interaction with negatively charged residues in proteins and by weak interactions provided by **B2**. In addition, *fac*-[Re(CO)_3_(**deeb**)**B2**]^+^ seems to interact preferentially with proteins and not with the gel matrix despite the presence of sodium dodecyl sulfate (SDS). In future applications, these alternative cationic complexes might be used alone or in combination with more traditional anionic compounds to generate counterion dye stains to improve the process.

## Highlights

1. Rhenium(I) complexes can be used to stain proteins
2. We synthesized and characterized a new cationic *fac*-[Re(CO)_3_(**deeb**)**B2**]^+^ complex
3. *fac*-[Re(CO)_3_(**deeb**)**B2**]^+^ properties are shown (UV-Vis, luminescence, and voltammetry)
4. Relativistic DFT, TD-DFT, and SOC-TDDFT calculations were performed
5. *fac*-[Re(CO)_3_(**deeb**)**B2**]^+^ can be used as a fluorescent protein dye in SDS-PAGE

## Introduction

Gel electrophoresis is a widely used molecular biology technique aimed to separate biomolecules with high resolution, which allows for multiple applications. Electrophoresis can be used to separate proteins based on their charge-to-mass ratio, shape, and size (non-denaturing gels) or based mainly on their size (denaturing gels) (Zewert and Harrington, 1993). In denaturing gels, sodium dodecyl sulfate (SDS), which coats the denatured protein through hydrophobic interactions, maintains the unfolded state due to SDS electrostatic repulsions. Consequently, negative charges provided by SDS dominate the total charge of proteins in an aqueous solution. This denaturing technique is known as sodium-dodecyl-sulfate polyacrylamide gel electrophoresis (SDS-PAGE) (Maizel, 2000; Samdin et al., 2020).

In order to observe proteins in the gel matrix, it is necessary to use staining methods. Some of these methods include colorimetric total protein stains, such as silver stain, considered one of the most sensitive procedures for detecting proteins; zinc staining, a negative method that stains the polyacrylamide gel only in regions where there are no proteins; and Coomassie brilliant blue (CBB; disulfonated triphenylmethane dye), one of the most used dyes (Rabilloud, 1990; Wirth and Romano, 1995; Patton, 2002; Sundaram et al., 2012). CBB binds to proteins via electrostatic interactions of the sulfonate (–SO_3_^−^) groups with protonated amino acids (–NH_3_^+^ or –NH^+^–) (i.e., lysine, arginine, or histidine) but also by hydrophobic interactions with the exposed aromatic residues (i.e., phenylalanine, tyrosine, and tryptophan) (Sundaram et al., 2012).

Fluorescent staining is an alternative technique that offers some exciting features. It has been stated that fluorescent staining presents higher linear quantitation ranges than colorimetric methods (Sundaram et al., 2012). Fluorescent protein gel staining is considered a technique that is sensitive, safe, and versatile. Although detection depends on a device providing a suitable excitation wavelength, common transilluminators, ordinarily present in molecular biology laboratories, can be used for this purpose. A device with a light-emitting diode or another source of filtered light, coupled with a filter for the emission, is sufficient to observe many of the UV-excitable dyes (Sundaram et al., 2012).

Some fluorescent stainings for proteins have been described. Epicocconone stain is an azaphilone that reacts with primary amines in proteins, generating a red fluorescent compound (Bell and Karuso, 2003). Nile red (Nile blue oxazone) is a phenoxazone that presents fluorescence enhancement in the presence of protein–SDS complexes, which makes it useful for SDS-PAGE (Sundaram et al., 2012). On the other hand, metal-based fluorescent dyes have also been reported. SYPRO^TM^ Ruby (a ruthenium-based compound whose formulation is held secret for commercial purposes) presents high sensitivity to proteins, which is equivalent to the silver staining method and independent of the presence of nucleic acids and lipopolysaccharides (Sundaram et al., 2012).

In the frame of the development of new fluorescent dyes to stain proteins, d^6^ transition metals can be considered, including Ru(II), Ir(III), and Re(I). d^6^ transition metals show useful photophysical properties, including visible-light absorption and emission, chemical stability, and low cytotoxicity (Fernandez-Moreira et al., 2010; Thorp-Greenwood et al., 2012; Carreño et al., 2019; Otero et al., 2019; Carreño et al., 2021). In addition, d^6^ transition-metal complexes have shown interesting biological applications not only as fluorophores but also in other areas such as selective cytotoxic complexes against cancer cells (Tian et al., 2017; Xu et al., 2018; Balaji et al., 2020; Li et al., 2020).

Regarding the d^6^ transition-metal complexes to stain proteins, Ru(II) bathophenanthroline disulfonate (RuBPS) is a luminescent complex that efficiently stains proteins in polyacrylamide gels (Rabilloud et al., 2001; Babak et al., 2020). This feature can be attributed to the bathophenanthroline disulfonate moiety, which allows water solubility and permits electrostatic interactions similar to that described for CBB (Graham et al., 1978; Berggren et al., 2000; Steinberg et al., 2000; Rabilloud et al., 2001). The bathophenanthroline disulfonate (BPS) moiety has been used to generate alternative d^6^ complexes to stain proteins in the polyacrylamide gels, including Ir(C,N)_2_(BPS) (Jia et al., 2012).

Regarding the use of Re(I) complexes, it has been reported that Re(I)-tetrazolato complexes *fac*-[Re(CO)_3_(N,N)(Tph)]^0/+/2−^, where Tph is 5-phenyl-tetrazolato, have been analyzed as luminescent dyes for proteins separated by electrophoresis (SDS-PAGE) (Fiorini et al., 2018). In that study, the authors explored different equatorial ligands, such as bathophenanthroline disulfonate (BPS) or bathocuproine disulfonate (BC), yielding *fac*-[Re(CO)_3_(BPS)(Tph)]^2−^ and *fac*-[Re(CO)_3_(BC)(Tph)]^2−^. As expected, these two anionic complexes successfully marked proteins separated by SDS-PAGE due to disulfonate groups (Fiorini et al., 2018). Although the presence of the disulfonate groups has been widely used to achieve the (dye-SO_3_^−^/^+^NH_3_–protein) interaction, as stated above, other strategies can also be used (Jin and Choi, 2004). Cationic dyes can also interact with proteins, presumably by electrostatic interactions with negatively charged residues (e.g., the presence of –COO^−^ in aspartate and glutamate) (Jin and Choi, 2004). The cationic complex *fac*-[Re(CO)_3_(BC)(Tph-Me)]^+^, where Tph-Me corresponds to 5-phenyl-4-methyl-tetrazolato, is also able to stain proteins but with reduced sensitivity in comparison with complexes harboring sulfonate groups. In order to improve these kinds of cationic complexes, new ancillary ligands have been reported as useful to modulate different properties. Since hydrophobic interactions, van der Waals forces, and hydrogen bonding can also contribute to the binding of the dye to proteins (Jin and Choi, 2004; Fiorini et al., 2018), the exploration of new ligands could allow obtaining alternative complexes to stain proteins, especially when considering the timesaving synthesis of sulfonated-free complexes (Fiorini et al., 2018). In this context, an ancillary ligand allowing for the formation of hydrophobic interactions, van der Waals forces, and/or hydrogen bonding with proteins could contribute to this development, even with cationic complexes.

**SCHEME 1:**
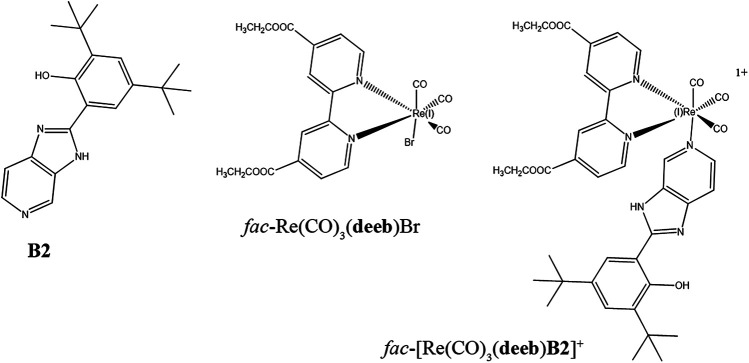
Chemical structure of **B2** [2,4-di-*tert*-butyl-6-(3H-imidazo[4,5-c]pyridine-2-yl)phenol], *fac*-Re(CO)_3_(**deeb**)Br, and *fac*-[Re(CO)_3_(**deeb**)**B2**]^+^.

The exploration of other luminescent molecules in biological applications has already been reported. **B2** [2,4-di-*tert*-butyl-6-(3H-imidazo[4,5-c]pyridine-2-yl)phenol] presents promising features regarding its use as a fluorophore for diverse biological applications. **B2** is an excellent luminescent cell dye for confocal microscopy, showing differential staining for the endoplasmic reticulum and the Golgi apparatus in epithelial cell lines (Carreno et al., 2016a; Llancalahuen et al., 2018) and staining walled cells, such as yeasts (*Candida albicans*), Gram-negative bacteria (*Salmonella enterica* and *Escherichia coli*), and Gram-positive bacteria (*Lactobacillus kunkeei*) for confocal microscopy (Carreno et al., 2016a; Berrios et al., 2018). Nevertheless, despite these remarkable properties, **B2** lacks sulfonate groups (–SO_3_^−^) and positively charged groups, suggesting that this compound cannot interact with proteins to permit an efficient protein staining. Therefore, we sought to generate a fluorescent dye for protein staining based on **B2**. In this work, we report the synthesis of a new complex *fac*-[Re(CO)_3_(**deeb**)**B2**]^+^ [where **deeb** is 4,4′-bis(ethoxycarbonyl)-2,2′-bipyridine], which was synthesized by replacing –Br from *fac*-Re(CO)_3_(**deeb**)Br (Hasselmann and Meyer, 1999b; Carreno et al., 2015; Carreño et al., 2017a) with **B2** (Scheme 1). The new *fac*-[Re(CO)_3_(**deeb**)**B2**]^+^ complex was synthesized and characterized using chemical techniques such as FTIR, ^1^H NMR, HHCOSY, mass spectrometry (EI-MS 894.3 M^+^), UV-Vis, emission, and cyclic voltammetry. We also performed a relativistic computational characterization to corroborate the experimental assignments and better understand the absorption and emission transitions. The complex showed luminescent emission with a large Stokes shift (*λ*_ex_ = 366 nm, *λ*_em_ = 625 nm), *τ* of 200 ns, and *φ* = 0.004. Finally, we found that *fac*-[Re(CO)_3_(**deeb**)**B2**]^+^ can fluorescently stain proteins separated by electrophoresis, presumably due to electrostatic interactions between the complex and negatively charged residues (e.g., –COO^−^) (provided by its cationic nature) but also plausibly by other kinds of interactions due to the presence of **B2** as the ancillary ligand. This work underlines that the synthesis of alternative complexes, i.e., cationic complexes lacking sulfonate groups, can also be designed to stain proteins. In future applications, these alternative cationic complexes might be used alone or in combination with more traditional anionic compounds to generate counterion dye stainings.

## Experimental and Theoretical Procedures

### Materials and Synthesis

All starting materials were purchased from Merck and Aldrich and used with no further purification. Acetonitrile (CH_3_CN) was dried in molecular sieves and purged under argon gas for electrochemical applications (Del Valle et al., 2009). Synthesis and characterization of rhenium(I) tricarbonyl complexes are described below.

#### Synthesis of B2 (2,4-Di-*tert*-butyl-6-(3H-imidazo[4,5-c]pyridine-2-yl)phenol)

The compound was obtained according to a procedure previously reported (Carreno et al., 2016a).

#### Synthesis of *fac*-Re(CO)_3_(**deeb**)**Br**

The complex was obtained as previously reported (Hasselmann and Meyer, 1999a). In this case, we used (Re(CO)_3_Br(THF))_2_ dimer and **deeb** as starting materials (Carreno et al., 2015).

#### Synthesis of *fac*-[Re(CO)_3_(**deeb**)**B2**]^+^

A suspension of *fac*-Re(CO)_3_(**deeb**)**Br** (in anhydrous THF) was added to Ag^+^(O_3_SCF_3_)^−^ (1:1 M ratio) under an inert atmosphere in the dark. The mixture was stirred at room temperature for 2–3 h under nitrogen. The precipitated AgBr byproduct was removed by filtration, and then **B2** (dissolved in anhydrous THF) (1:1 M ratio) was added before refluxing for 5 h under nitrogen (Carreno et al., 2015). The solution was concentrated using a rotary evaporator, and the solid residue was dissolved in ethanol. Excess NH_4_PF_6_ was added, and the mixture was stirred for 24 h. The solid was precipitated, collected, and recrystallized from ethanol/diethyl ether (1:1, v/v). Yield = around 70.0%. FTIR (cm^−1^): 3331 and 3125 (νOH), 2034 and 1925 (νCO), 1777 (νCO), 832 (νPF_6_^−^). ESI-MS Calcd (found) for ReC_39_H_41_N_5_O_8_: m/z ^+^ 893.96 (894.3). ^1^H NMR (400 MHz, CD_3_CN): *δ* = 1.35 [s, 9H], 1.42 [s, 9H], 5.59 [s, 2H, –NH_2_], 6.45 [d; J = 5.5 Hz; 1H], 7.28 [d; J = 1.5 Hz; 1H], 7.43 [s, 1H], 7.51 [s, 1H], 7.53 [s, 1H], 7.77 [dd; J = 5.5; 7.9 Hz; 2H], 8.11 [s, 1H], 8.28 [dd; J = 5.6; 7.9 Hz; 2H], 8.39 [d; J = 8.3 Hz; 2H], 8.91 [s, 1H], 9.21 [d; J = 5.6 Hz; 2H], 12.54 [s, –OH]. UV-Vis (acetonitrile, room temperature): *λ* = 338 nm.

### Physical Measurements

FTIR spectra were obtained on a Bruker Vector-22 FT-IR spectrophotometer. The ^1^H NMR spectra for **B2**, *fac*-Re(CO)_3_(**deeb**)**Br**, and *fac*-[Re(CO)_3_(**deeb**)**B2**]^+^ were recorded on a Bruker AVANCE 400 spectrometer at 400 MHz at 25°C. The sample was dissolved in deuterated solvents (DMSO-_d6_ or acetonitrile-_d3_) using tetramethylsilane as an internal reference. The UV-Vis absorption spectrum for *fac*-[Re(CO)_3_(**deeb**)**B2**]^+^ was recorded using a Varian Cary 60 UV-Vis spectrophotometer with a resolution of 1 nm. The steady-state photoluminescence spectrum for *fac*-[Re(CO)_3_(**deeb**)**B2**]^+^ was measured on an ISS K2 fluorometer. The samples were sparged with argon-saturated acetonitrile for 30 min and excited at *λ* ∼445 nm. The intensity was integrated for 0.5 s with a 2 nm resolution. The photoluminescence quantum yield was measured through comparative actinometry using [Ru(2,2′-bipyridine)_3_][PF_6_]_2_ in acetonitrile (φ_em_ = 0.06) as a quantum yield standard (Bond et al., 2002; Hess et al., 2017). Photoluminescence lifetime measurements were performed using a PTI nitrogen dye laser with excitation centered around 445 nm. Decays were monitored at the photoluminescence maximum and averaged over 180 scans.

The mass spectra of *fac*-[Re(CO)_3_(**deeb**)**B2**]^+^ were analyzed with UPLC Xevo G2 Q-TOF (Waters) (high resolution mass spectrometer).

For electrochemical experiments, the working solution contained 0.01 mol/L of the respective compound (i.e., **B2**, *fac*-Re(CO)_3_(**deeb**)Br, and *fac*-[Re(CO)_3_(**deeb**)**B2**]^+^) with 0.1 mol/L tetrabutylammonium hexafluorophosphate (TBAPF_6_, supporting electrolyte) in anhydrous acetonitrile (CH_3_CN). Before each experiment, the working solution was purged with high purity argon, and an argon atmosphere was maintained during the whole experiment. A polycrystalline, non-annealed platinum disc (2 mm diameter) was used as the working electrode. A platinum gauze of a large geometrical area, separated from the main cell compartment by a fine sintered glass, was used as the counter electrode (Del Valle et al., 2012; Ramírez et al., 2016). All potentials quoted in this paper are referred to as Ag/AgCl electrodes in tetramethylammonium chloride to match the potential of a saturated calomel electrode (SCE) at room temperature. All electrochemical experiments were performed at room temperature on a CHI900B bipotentiostat interfaced to a PC running CHI 9.12 software that allowed for experimental control and data acquisition.

### Computational Details

All the calculations were performed using the Amsterdam Density Functional (ADF) code (Te Velde et al., 2001). The scalar relativistic and spin–orbit coupling effects were incorporated using the two-component Hamiltonian with the zeroth-order regular approximation (ZORA Hamiltonian) (Lenthe et al., 1993; Anton et al., 2004). The ground and first excited triplet state geometries were optimized at the B3LYP/TZ2P level of theory (Kim and Jordan, 1994; Stephens et al., 1994; Van Lenthe and Baerends, 2003). Implicit solvation effects on the geometry optimization and optical properties were considered using a dielectric continuum model (COSMO) with acetonitrile as a solvent (Van Lenthe and Baerends, 2003; Bickelhaupt and Baerends, 2007). The molecular structure was considered without terminal methyl groups in the bipyridine moiety to avoid convergence problems due to the group rotation. This procedure has no impact on the studied luminescent phenomena.

Scalar relativistic time-dependent density functional theory (SR-TDDFT) was used to calculate the absorption spectra for the 30 singlet and 30 triplet excitations, which were subsequently used as the basis for the self-consistent two-component spin–orbit coupling TDDFT (SOC-TDDFT) within the ZORA Hamiltonian (Wang and Ziegler, 2005; Krause and Klopper, 2015).

### Protein Visualization

**Bacterial strains.** We worked with *Salmonella enterica* subspecies *enterica* serovar Typhimurium ATCC 14028s (simply called Typhimurium).

**Bacterial culture.** Bacteria were cultured under standard laboratory conditions (i.e., shaking at 37°C) in Luria–Bertani broth (Bacto tryptone, 10 g/L; NaCl, 5.0 g/L; yeast extract, 5 g/L) (Nevermann et al., 2019). Bacteria were harvested at the stationary phase (OD_600_ = 1.4).

**Protein obtention.** Bacteria were centrifuged for 10 min at 5400 ×*g* (4°C). The supernatant was discarded, and the pellet was washed three times with buffer Tris-HCl 10 mM pH 8.0 (Nevermann et al., 2019). Bacteria were finally resuspended in Tris-HCl 10 mM pH 8.0, 1 mM phenylmethylsulfonyl fluoride (PMSF), and 1 mM ethylenediaminetetraacetic acid (EDTA) before being sonicated on ice for 100 s. Proteins were stored at −80°C until use.

**Determination of protein concentration.** The total protein concentration was determined by the BCA^TM^ Protein Assay Kit (Thermo Scientific) according to the manufacturer’s instructions.

**SDS-PAGE.** Polyacrylamide gels (12.5%) (Nevermann et al., 2019) were prepared in BioRad^TM^ chambers (separation gel: 2.415 μl [acrylamide 30%:bisacrylamide 0.8%], 1.320 μl buffer Tris-HCl 1.5 M pH 8.8, 52.5 μl sodium dodecyl sulfate [SDS] 10%, 955 μl distilled water, 150 μl ammonium persulfate [PSA], and 7.5 μl N,N,N′,N′-tetramethyl-ethylenediamine [TEMED]; stacking gel: 312 μl [acrylamide 30%:bisacrylamide 0.8%], 450 μl buffer Tris-HCl 0.5 M pH 6.8, 1.040 μl distilled water, 50 μl PSA, and 3.75 μl TEMED). The electrophoresis chamber was filled with running buffer (1.44% glycine, 0.3% Tris, and 0.1% SDS). Then, proteins were mixed with one volume of load buffer (187.5 mM Tris-HCl pH 6.8, 6% SDS, 30% glycerol, 0.03% bromophenol blue, and 15% *β*-mercaptoethanol), before being incubated at 98°C for 5 min. A total of 100 μg proteins were loaded in the respective lanes. Electrophoresis was performed at a constant voltage of 50 V until the sample reached the separation gel, where the voltage was increased to 100 V.

**Protein staining.** The gel was incubated for 30 min at room temperature with distilled water prior to incubating it in a solution of **B2**, *fac*-Re(CO)_3_(**deeb**)Br, or *fac*-[Re(CO)_3_(**deeb**)**B2**]^+^ (400 μg/ml in DMSO) for 60 min at room temperature covered with an aluminum foil. Finally, the gel was washed with either distilled water for 10 min or DMSO 25% in distilled water for 12 h under gentle shaking. Stained proteins were observed in a UV transilluminator ECX-20M (λ_exc_ = 312 nm).

## Results and Discussion

### Characterization of *fac*-[Re(CO)_3_(deeb)B2]^+^

As stated above, the *fac*-Re(CO)_3_ (**deeb**)**Br** complex was obtained as previously reported (Hasselmann and Meyer, 1999a; Hasselmann and Meyer, 1999b). Nevertheless, in this work, *fac*-Re(CO)_3_(**deeb**)**Br** was synthesized by an alternative method based on the reaction of the **deeb** ligand with the (Re(CO)_3_Br(THF))_2_ dimer (Carreno et al., 2015). On the other hand, **B2** was synthesized as reported (Carreno et al., 2016a). The ^1^H NMR of both *fac*-Re(CO)_3_(**deeb**)**Br** and **B2** showed expected values (Supplementary Figures S1, 2 in the Supplementary Material). In order to synthesize *fac*-[Re(CO)_3_(**deeb**)**B2**]^+^, we used a previously reported procedure (Carreno et al., 2015), obtaining high yield (around 70%). Characteristic constants of *fac*-[Re(CO)_3_(**deeb**)**B2**]^+^ (i.e*.*, molecular weight, yield, and solid color) are shown in Supplementary Table S1 in the Supplementary Material. The mass spectrometric analysis (FOB) of *fac*-[Re(CO)_3_(**deeb**)**B2**]^+^ (ReC_39_H_41_N_5_O_8_^1+^) showed a central fragment at m/z = 894.3, in agreement with the expected value for the molecular ion (it aligns with the Re^+^ complex without PF_6_^−^). The isotope structure is consistent with the rhenium composition (Supplementary Figure S3 in the Supplementary Material). The FTIR spectrum, along with both 1D and 2D ^1^H NMR assays (see below), was used to confirm the *fac*-[Re(CO)_3_(**deeb**)**B2**]^+^ structure. In the FTIR spectrum, the range around 3500–3000 cm^−1^ presented bands that are generally considered symmetric and asymmetric, due to νOH and/or νNH vibrations at 3331 cm^−1^ in the **B2** moiety (Supplementary Figure S4 in the Supplementary Material). We assigned the bands at 3125 cm^−1^ (aromatic νCH) and 2963 cm^−1^ and 2873 cm^−1^ (νCH). On the other hand, regarding the presence of carbonyl groups in the *fac*-[Re(CO)_3_(**deeb**)**B2**]^+^ complex, only two bands were observed at 2034 cm^−1^ and 1925 cm^−1^ in the FTIR spectrum. This result can be explained by a convolution of facial carbonyls due to a local symmetry loss. Thus, the carbonyl stretching was affected by the d*π* electron density due to the ancillary ligand (**B2**). On the other hand, the *π*–back bonding effect in the carbonyls (–CO) also was observed. This effect can be explained by delocalization of electron density from the rhenium(I) metal to the carbonyl π*-orbitals, resulting in a lowered CO stretching frequency, compared to free CO (2143 cm^−1^) (Bistoni et al., 2016). The 1733 cm^−1^ mode was assigned to the carbonyl group of the ester groups in the **deeb** ligand. Other important modes appeared at 1617 cm^−1^, and 1578 cm^−1^ (νCN) and 1430 cm^−1^ (νCC) in the aromatic rings, and a signal at 832 cm^−1^ was assigned to the PF_6_^−^ anion.

The *fac*-[Re(CO)_3_(**deeb**)**B2**]^+^ complex was also characterized by its ^1^H NMR spectra (1D and 2D) in acetonitrile-_d3_ (for proton numbering, see Supplementary Scheme S1; for ^1^H NMR, see Supplementary Figure S5; for the expanded aromatic region, see Supplementary Figure S6 in the Supplementary Material). The **deeb** protons in the *fac*-[Re(CO)_3_(**deeb**)**B2**]^+^ complex were assigned at 9.40 ppm (H1′, d, J = 5.7 Hz), 9.13 ppm (H1", d, J = 5.7 Hz), 8.18 ppm (H2′, dd, J = 5.7 and 1.2 Hz), 8.09 ppm (H2", dd, J = 5.6 and 1.4 Hz), 8.93 ppm (H3′, s), and 8.79 ppm (H3", s). These results indicate that the protons of the **deeb** moiety are diastereotopic, in contrast with what was observed with the **deeb** ligand in the *fac*-Re(CO)_3_(**deeb**)Br complex (Supplementary Figure S1 in the Supplementary Material), in which the protons have the same chemical shift (homotopic). In the case of the **B2** moiety in the *fac*-[Re(CO)_3_(**deeb**)**B2**]^+^ complex, we observed a general displacement to higher fields in comparison with free **B2** (compare Supplementary Figure S2 with Supplementary Figure S5 in the Supplementary Material). The assignment was as follows: 8.54 ppm (H3), 7.97 ppm (H1), 7.36 ppm (H2), 7.55 ppm (H4), and 7.45 ppm (H5). The –OH and –NH groups were observed at 12.75 ppm and 5.96 ppm, respectively. These signals disappeared with D_2_O exchange, confirming the assignation (Supplementary Figure S7 in the Supplementary Material).

On the other hand, the resonances of aromatic protons found in the **deeb** ligand in *fac*-[Re(CO)_3_(**deeb**)**B2**]^+^ showed lower values compared to those of the corresponding *fac*-Re(CO)_3_(**deeb**)Br precursor, suggesting that **B2** may be a weaker electron-donating group compared with −Br or may exert a withdrawing effect over the Re(I) core (compare Supplementary Figures S1 **and** 5 in the Supplementary Material).

Other signals were assigned at 1.25 ppm and 1.35 ppm corresponding to the *tert*-butyl protons in **B2** and at 4.39 ppm (q, –CH_2_–) and 1.40 ppm (t, –CH_3_–) corresponding to the **deeb** moiety (Supplementary Figure S5 in the Supplementary Material). The HHCOSY experiment confirmed the assignments (Supplementary Figure S8 in the Supplementary Material). The FTIR, MS^+^, and ^1^H NMR assays, together, confirmed the proposed structure.

The absorption spectrum of *fac*-[Re(CO)_3_(**deeb**)**B2**]^+^ in acetonitrile was recorded at room temperature. The *fac*-[Re(CO)_3_(**deeb**)**B2**]^+^ complex showed the expected absorption bands for this kind of compound (Carreno et al., 2015; Carreno et al., 2016b; Carreño et al., 2017b) centered around 284 nm and 335 nm. In addition, we observed a third, not very intense, band centered around 405 nm (Supplementary Figure S9 in the Supplementary Material). The composition of these bands is discussed below. This spectrum was blue-shifted relative to the *fac*-Re(CO)_3_(**deeb**)Br precursor, which showed intense adsorption bands centered at 312 nm and 419 nm, as reported previously (Hasselmann and Meyer, 1999a; Hasselmann and Meyer, 1999b). This blue shifting may be attributed to the π-acidic nature of **B2** compared with Br present in *fac*-Re(CO)_3_(**deeb**)Br, although the difference in electronic effect between **B2** and −**Br** may play a more significant role. The blue shifting has already been reported for similar compounds with ancillary ligands presenting intramolecular hydrogen bonds (Carreno et al., 2015; Carreno et al., 2016b; Carreño et al., 2017b).

**FIGURE 1:**
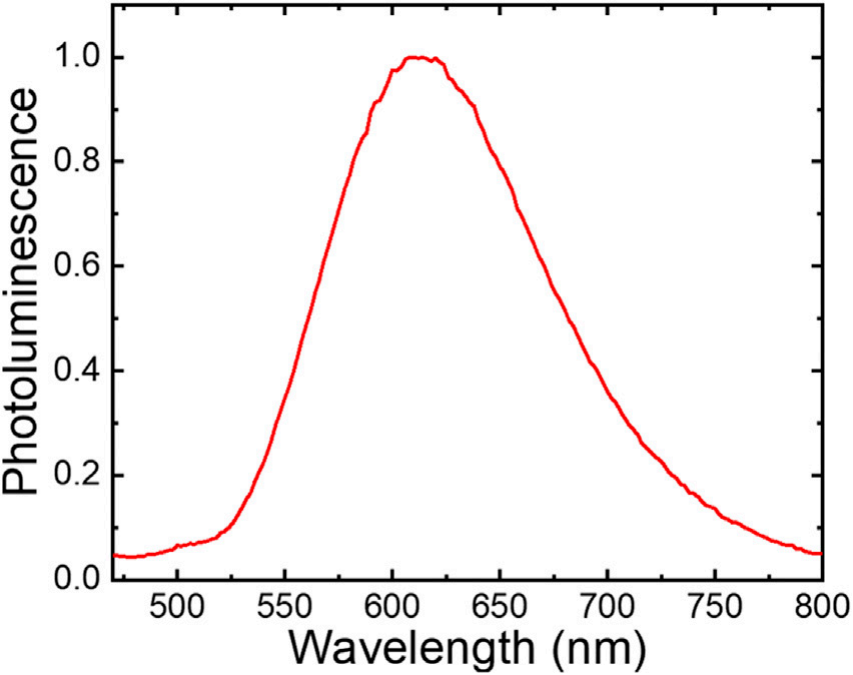
Photoluminescence spectra of *fac*-[Re(CO)_3_(**deeb**)**B2**]^+^ in acetonitrile (λ_exc_ = 445 nm).

Steady-state 445 nm light excitation of *fac*-[Re(CO)_3_(**deeb**)**B2**]^+^ in acetonitrile resulted in room temperature photoluminescence with a broad band centered around λ_max_ = 615 nm (Figure 1). Thus, a large Stokes-like shift was observed, consistent with a large change in the dipole moment between the ground and excited states (emission lifetime = 200 ns, φ = 0.004). These emission processes are characteristic of rhenium(I) tricarbonyl complexes and agree with the long-lived ^3^MLCT excited states (Thorp-Greenwood et al., 2016; Malecka et al., 2020; Maron et al., 2020; Carreño et al., 2021).

Table 1 summarizes the photophysical properties of **B2** and the new complex *fac*-[Re(CO)_3_(**deeb**)**B2**]^+^. We attribute the low quantum yield of *fac*-[Re(CO)_3_(**deeb**)**B2**]^+^, compared with its precursor *fac*-Re(CO)_3_(**deeb**)Br in the excited states (quantum yield = 0.030, τ = 30 ns (Hasselmann and Meyer, 1999a)), to intramolecular electron transfer in the ^3^MLCT excited state initiated by the presence of a redox-active phenol group (see below) coupled to proton transfer from phenol to nitrogen in the **B2** moiety. Such intramolecular quenching has been previously reported through flash quench experiments of a ruthenium bipyridine complex (Zhang et al., 2011; Nomrowski and Wenger, 2015; Lennox et al., 2017). The proton-coupled electron transfer has also been observed for rhenium(I) tricarbonyl complexes in intermolecular interactions (Manbeck et al., 2016; Prado et al., 2016; Pannwitz and Wenger, 2019). The presence of the intramolecular hydrogen bond (IHB) in **B2** could also be contributing to the relatively low quantum yield in the *fac*-[Re(CO)_3_(**deeb**)**B2**]^+^ complex. It has been reported for similar Re(I) complexes harboring ancillary ligands with an IHB that the quantum yields can be improved by disrupting the IHB with a mixture of D_2_O/CH_3_CN (Carreno et al., 2016b). Thus, it would be possible to achieve similar effects by synthesizing a **B2** derivative lacking −OH involved in the IHB. Nevertheless, this hypothesis remains to be experimentally tested.

**TABLE 1:** Photophysical properties of the uncoordinated **B2** (Carreno et al., 2016a) ligand and *fac*-[Re(CO)_3_(**deeb**)**B2**]^+^.

Compound	τ (ns)	Quantum yield	k_r_ (M^−1^ s^−1^)	k_nr_ (M^−1^ s^−1^)
**B2**	<10^a^	0.21	2.1 × 10^7^ ^b^	7.9 × 10^7^ ^b^
*fac*-[Re(CO)_3_(**deeb**)**B2**]^+^	200	0.004	2.0 × 10^4^	4.98 × 10^6^

^a^ Lifetime was faster than the instrument response.

^b^ These values are approximated.

### Electrochemical Behavior

**FIGURE 2:**
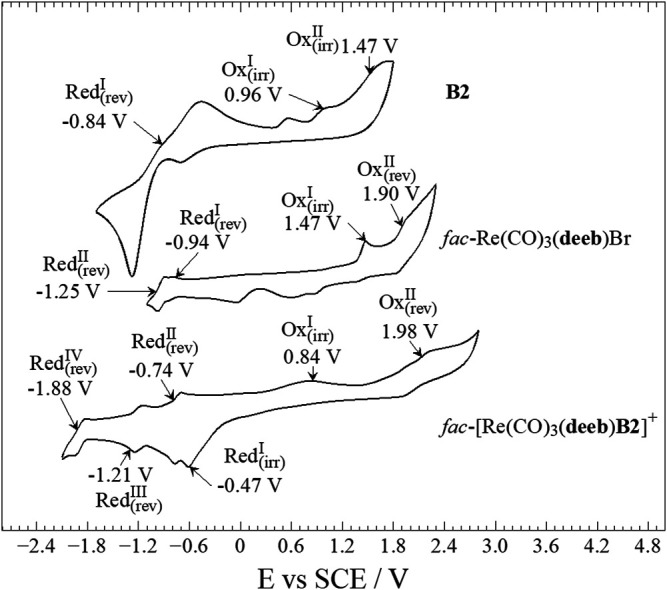
Compared electrochemical response of **B2**, *fac*-Re(CO)_3_(**deeb**)Br, and *fac*-[Re(CO)_3_(**deeb**)**B2**]^+^. Interphase: Pt|1.0 × 10^–2^ M of compound + 1.0 × 10^–1^ M TBAPF_6_ in anhydrous CH_3_CN.

In order to further characterize *fac*-[Re(CO)_3_(**deeb**)**B2**]^+^, we compared its electrochemical behavior with that of its precursor *fac*-Re(CO)_3_(**deeb**)Br (Carreno et al., 2015) and its ancillary ligand **B2** (Carreno et al., 2016a). Our analysis is shown in Figure 2.

Regarding the electrochemical response of *fac*-Re(CO)_3_(**deeb**)Br, we found irreversible oxidation of rhenium at E_p_ + 1.47 V, according to the previously reported value (Hasselmann and Meyer, 1999a; Carreno et al., 2015). In the present study, we found a second reversible oxidation at E_½_ + 1.90 V (Figure 2). This process was observed due to an increase in the potential limit explored. These oxidation processes follow an electrochemical–chemical–electrochemical reaction (ECE) mechanism (Czerwieniec et al., 2005; Canales et al., 2017; Lindley et al., 2018) with the following steps: 1) first electrochemical one-electron oxidation of the rhenium center, Re^I→II^, followed by 2) an intramolecular Re/ligand redox process, Re^II→I^, with the ligand (Br) substitution by solvent molecules, and ending with 3) a second electrochemical one-electron oxidation of the rhenium center, Re^I→II^.

In the case of *fac*-[Re(CO)_3_(**deeb**)**B2**]^+^, two oxidations were found (see the working window study in Supplementary Figures S10 **and** 11 and in the Supplementary Material). The first oxidation was determined to be irreversible at E_p_ + 0.84 V and possibly corresponds to one of the irreversible processes in the **B2** moiety; the other oxidation process in the **B2** moiety may not take place or be masked due to the intense oxidation current from the second oxidation in *fac*-[Re(CO)_3_(**deeb**)**B2**]^+^. This second oxidation was reversible at E_½_ + 1.98 V and most likely corresponds to the Re^I→II^ process (Figure 2). Thereby, the presence of the **B2** ligand in *fac*-[Re(CO)_3_(**deeb**)**B2**]^+^, compared with *fac*-Re(CO)_3_(**deeb**)Br, produces a strong potential shift for the first step on the ECE mechanism 1) Re^I→II^ to a higher energy value. This result agrees with the proposed electron-withdrawing nature of the **B2** moiety in *fac*-[Re(CO)_3_(**deeb**)**B2**]^+^ (see the NMR and absorption study), which decreases the possibility (i.e*.*, it increases the required potential) to accomplish the rhenium center oxidation.

Concerning the reduction processes observed, it has been reported that the free **deeb** ligand presents a reversible reduction process at E_½_ −0.90 V, whereas free **B2** exhibits a strong quasi-reversible reduction process at E_½_, −0.84 V vs. SCE (Carreno et al., 2015; Carreno et al., 2016a). It is worth mentioning that the reported analyses were carried out under the same experimental condition used in this study (Figure 2). As previously reported, the **deeb** ligand reduction shifts from −0.90 V to −0.94 V in *fac*-Re(CO)_3_(**deeb**)**Br**, with reversible rhenium reduction Re^I→0^ appearing at E_½_ −1.25 V (Wallace and Rillema, 1993; Lo et al., 2004; Zeng et al., 2012; Carreno et al., 2015). In the case of *fac*-[Re(CO)_3_(**deeb**)**B2**]^+^, we found four reduction processes determined after the working window potential study (Supplementary Figures S10, 11). The first reduction process at Ep −0.47 V can be attributed to the **B2** moiety since this is the more intense and irreversible process. On the other hand, the reversible reduction at E_½_ −0.74 V found for *fac*-[Re(CO)_3_(**deeb**)**B2**]^+^ was attributed to the **deeb** ligand due to its similarity to that previously reported in potential terms (around E_½_ −0.90 V) (Carreno et al., 2015).

Finally, both reversible reductions at E_½_ −1.21 and −1.88 V correspond to Re^I→0^ but following different alternate pathways. At E_½_ −1.21 V, the rhenium reduction takes place, followed by intramolecular reduction and elimination of **B2** in its reduced form, **B2** ^−^:

$$fac-{\left[Re^{I}{\left(CO\right)}_{3}\left(\mathrm{deeb}\right)B2\right]}^{+}+e^{-}⇌fac-{\left[Re^{0}{\left(CO\right)}_{3}\left(\mathrm{deeb}\right)B2\right]}^{0}\;\;\;E_{1/2}:\;-1.21\;V\;\left(i\right),$$

$$fac-{\left[Re^{0}{\left(CO\right)}_{3}\left(\mathrm{deeb}\right)B2\right]}^{0}\to fac-{\left[Re^{I}{\left(CO\right)}_{3}\left(\mathrm{deeb}\right)\right]}^{+}+B2^{-}\;\;\;slow\;\left(ii\right).$$

Since (ii) is slow, the *fac*-[Re(CO)_3_(deeb)B2]^−^ species may suffer a second reduction process at a more negative potential. Thus, a second reduction takes place at E_½_ −1.88 V:

$$fac-{\left[Re^{0}{\left(CO\right)}_{3}\left(\mathrm{deeb}\right)B2\right]}^{0}+e^{-}⇌fac-{\left[Re^{0}{\left(CO\right)}_{3}\left(\mathrm{deeb}\right)B2\right]}^{-}\;\;\;E_{1/2}:\;-1.88\;V\;\left(iii\right),$$

$$fac-{\left[Re^{0}{\left(CO\right)}_{3}\left(\mathrm{deeb}\right)B2\right]}^{-}\to fac-{\left[Re^{I}{\left(CO\right)}_{3}\left(\mathrm{deeb}\right)\right]}^{0}+B2^{-}\;\;\;fast\;\left(iv\right).$$

These processes correspond to a typical electrochemical–electrochemical–chemical (EEC) mechanism, previously reported for similar rhenium complexes, with **B2** elimination as the chemical step (Manbeck et al., 2015; Abdellah et al., 2017). Thus, for *fac*-[Re(CO)_3_(**deeb**)**B2**]^+^, Re^I→0^ takes place at −1.21 V, while the same process occurs at −1.25 V in the *fac*-[Re(CO)_3_(**deeb**)]Br precursor. Therefore, this result supports the electron-withdrawing nature of **B2** on the metallic core. When **B2** is present in the *fac*-[Re(CO)_3_(**deeb**)**B2**]^+^ complex, the reduction shifts to less negative potential values (i.e., the reduction requires less energy to take place).

Finally, Table 2 presents an electrochemical characterization summary for *fac*-[Re(CO)_3_(**deeb**)**B2**]^+^, including the electrochemical processes found, reversibility, whether they are controlled by mass transport or not (determined by a scan-rate study presented, see Supplementary Figure S11 and Supplementary Table S2 in the Supplementary Material), and the corresponding designation.

**TABLE 2:** Electrochemical characterization summary of *fac*-[Re(CO)_3_(deeb)B2]^+^.

	Potential (V)	Reversibility	Diffusion control?	Designation
Ox^I^	Ep +0.84	Irreversible	Yes	**B2** oxidation
Ox^II^	E_½_ +1.98	Reversible	Yes	Re^I→II^, with **B2** elimination
Red^I^	Ep −0.47	Irreversible	Yes	**B2** reduction
Red^II^	E_½_ −0.74	Reversible	Yes	**deeb** reduction
Red^III^	E_½_ −1.21	Reversible	Yes	Re^I→0^
Red^IV^	E_½_ −1.88	Reversible	Yes	Re^I→0^, with **B2** ^−^ elimination

### Quantum Chemistry

Although we were unable to obtain the crystal of *fac*-[Re(CO)_3_(**deeb**)**B2**]^+^, the computational studies were based on X-ray data obtained from a similar complex previously reported, i.e., [*fac*-Re(CO)_3_(4,4′-dimethyl-2,2′-bpy)(*E*-2-((3-amino-pyridin-4-ylimino)-methyl)-4,6-di-*tert*-butylphenol)^+^] (Carreño et al., 2017b).

The most important optimized bond distances and angles of *fac*-[Re(CO)_3_(deeb)B2]^+^ are summarized in Supplementary Tables S3, 4, and 5 in the Supplementary Material. For atom numbering, see Supplementary Figure S13 in the Supplementary Material. As inferred by our results, the three carbonyls of *fac*-[Re(CO)_3_(**deeb**)**B2**]^+^ are distributed with facial coordination according to the observed FTIR spectra (Supplementary Figure S4 in the Supplementary Material). The coordination is completed by the deeb ligand in the equatorial plane and the B2 ligand in the axial plane.

**FIGURE 3:**
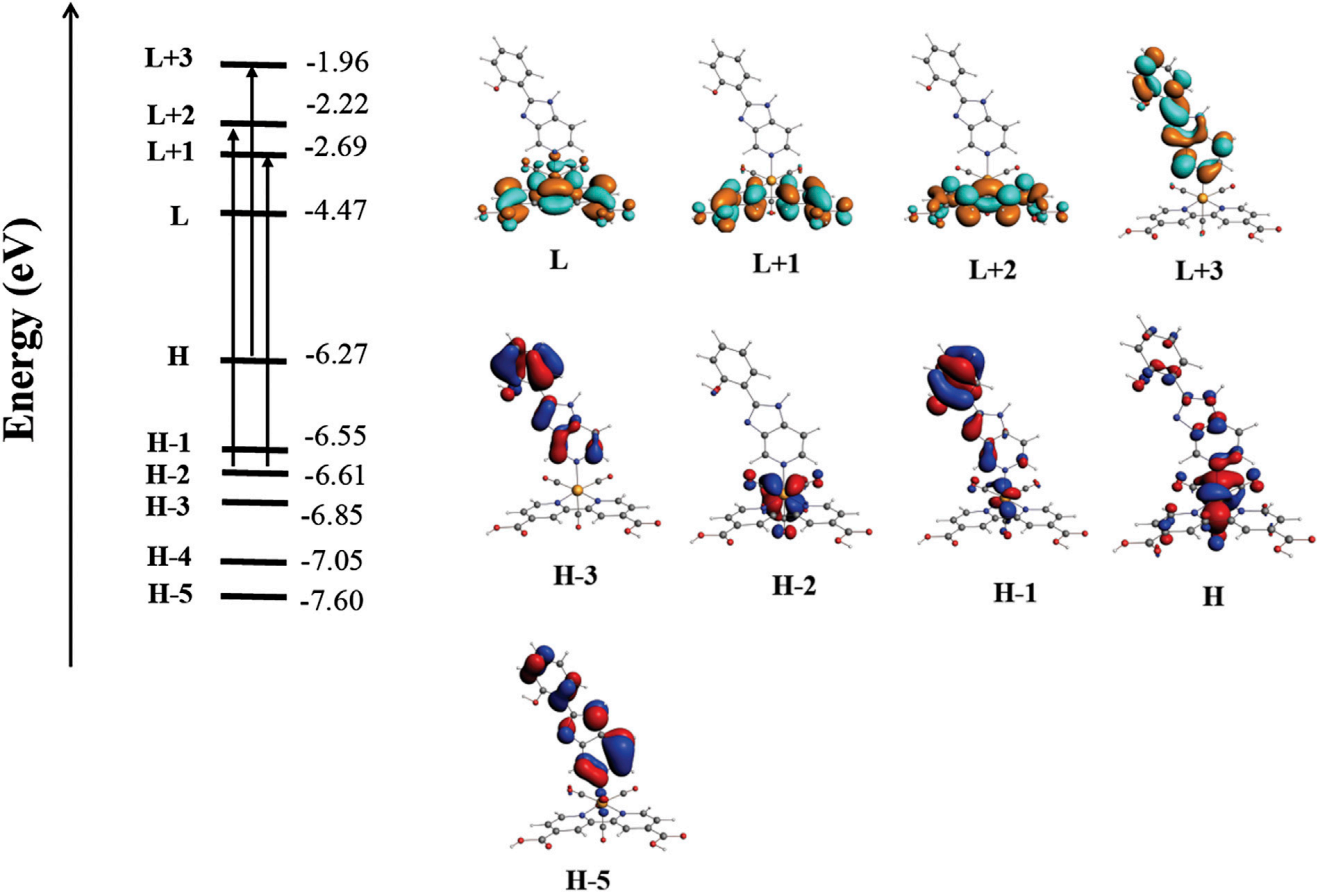
Qualitative molecular orbital diagram showing the most important electronic transitions involved in the absorption spectra of *fac*-[Re(CO)_3_(**deeb**)**B2**]^+^.

Time-dependent density functional theory (R-TDDFT) (Macleod‐Carey et al., 2018) calculations were performed to complement our analysis to elucidate and assign the calculated electronic transitions (Carreno et al., 2017; Rojas-Poblete et al., 2018). The calculated UV-Vis spectra in acetonitrile showed two principal absorption bands centered in the range 330–340 nm and 375 nm, in agreement with the experimental data. These bands were assigned as a combination of π → π* and MLCT electronic transitions (Table 3, Supplementary Figure S14 in the Supplementary Material). The isosurfaces of the involved molecular orbitals are shown in Figure 3. In this analysis, we can observe that the molecular orbitals of both the **deeb** and ancillary ligands are involved in the electronic transition. As described above, we experimentally obtained two typical bands around 284 and 335 nm. The band obtained at 284 nm corresponds to the predicted band at 311 nm, where H-1 → L + 3 corresponds to p*π*(**B2**) to pπ*(**B2**) (intraligand transition) and H-3 → L + 2 corresponds to p*π*(**B2**) to pπ*(**deeb**) (interligand transition). The band obtained experimentally around 335 nm presents a mixed composition according to our calculations. In the computed spectra, we obtained three bands with the higher harmonic oscillator (f) found in the range of 335–375 nm, which correspond to the experimental band of 335 nm. The predicted band at 335 nm is composed of H → L + 3 (100%) corresponding to [d*π*(Re) and p*π*(**B2**)] to pπ*(**B2**). The calculated band at 346 nm is composed of H-5 → L (80%) and H-2 → L + 2 (20%) corresponding to d*π*(**B2**) to pπ*(**deeb**) (interligand transition) and dπ(Re) to pπ*(**deeb**) (MLCT), respectively. The calculated band at 375 nm is composed of H-2 → L + 1 (90%) corresponding to d*π*(Re) to pπ*(**deeb**) (MLCT). The less intense band experimentally observed around 405 nm corresponds to the calculated band at 415 nm, which presents a low harmonic oscillator (Table 3).

**TABLE 3:** Calculated absorption bands considering the solvent effect (acetonitrile).

Molecule	*λ* (nm)	f	Origin	Assignment
*fac*-[Re(CO)_3_(**deeb**)**B2**]^+^	311	0.111	H − 1 → L + 3 (60%); H−3 → L + 2 (40%)	π → π*
	331	0.263	H − 2 → L + 2 (75%); H−5 → L + 2 (20%)	MLCT
	335	0.631	H → L + 3 (100%)	MLCT
	346	0.458	H − 5 → L (80%); H − 2 → L + 2 (20%)	π → π* MLCT
	375	0.203	H − 2 → L + 1 (90%)	MLCT
	415	0.130	H → L + 1	MLCT

Finally, the unoccupied molecular orbitals immediately around the LUMO are centered in both **deeb** and **B2**, suggesting that the ligands are directly involved in the observed emission.

To better understand the emission nature of *fac*-[Re(CO)_3_(**deeb**)**B2**]^+^, we carried out calculations at the SOC-TDDFT level of theory considering the solvent (acetonitrile) effect. This calculation also analyzes the three substrates derived from the excited triplet state due to spin–orbit coupling, estimating the zero-field splitting (ZFS) parameter and emission time. The calculated emission band was localized at 665 nm (Table 4) and assigned as ^3^LMCT involving a π* orbital located on **deeb** and the non-bonding, metal-centered d-orbitals. The calculated ZFS also corroborates this assignment and evidences the critical role of the metal-centered orbitals and spin–orbit coupling in the luminescent properties of *fac*-[Re(CO)_3_(**deeb**)**B2**]^+^. This behavior is consistent with that reported in previous studies using similar substituted bipyridines as ligands (Nastasi et al., 2015; Kia and Safari, 2016; Hostachy et al., 2017; Kang et al., 2017) and confirms that the emission occurs in the visible spectrum region.

**TABLE 4:** Calculated emission band considering solvent effects (acetonitrile).

Molecule	Calculated *λ*_em_ (nm)	ZFS	f	τ (s)
*fac*-[Re(CO)_3_(**deeb**)**B2**]^+^	665	59.5	0.96 × 10^–3^	2 × 10^–3^

### Luminescent Staining of Proteins

As previously described, rhenium(I) tricarbonyl complexes harboring disulfonated phenanthroline-derivative ligands have been reported as useful to stain proteins separated by SDS-PAGE. Nevertheless, the use of rhenium(I) complexes lacking these ligands has been less studied to this end (Fiorini et al., 2018). In this sense, we explored whether the cationic complex *fac*-[Re(CO)_3_(**deeb**)**B2**]^+^, its respective precursor *fac*-Re(CO)_3_(**deeb**)Br, or the free ligand **B2** was able to stain proteins resolved by SDS-PAGE. To that aim, a total extract of bacterial proteins was obtained before resolving them in SDS-PAGE, as indicated in *Protein Visualization in Methods*. Gels were washed with distilled water and then incubated with a solution of *fac*-[Re(CO)_3_(**deeb**)**B2**]^+^, *fac*-Re(CO)_3_(**deeb**)Br, or **B2** (400 μg/ml in DMSO as solvent) for 60 min at room temperature covered with an aluminum foil. Gels were washed with either distilled water (10 min) or DMSO (25% in distilled water, 12 h) and then examined with a UV transilluminator ECX-20M (*λ* = 312 nm).

**FIGURE 4:**
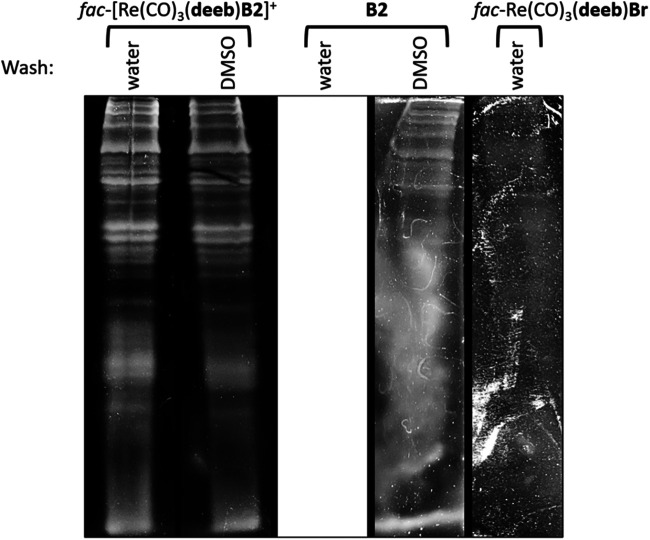
Total proteins were extracted and resolved in SDS-PAGE before being stained with *fac*-[Re(CO)_3_(**deeb**)**B2**]^+^, **B2**, or *fac*-Re(CO)_3_(**deeb**)Br for 60 min at room temperature. Gels were then washed with either distilled water (10 min) or DMSO 25% (12 h). Stained proteins were observed in a UV transilluminator ECX-20M (*λ* = 312 nm).

As shown in Figure 4, we observed that *fac*-[Re(CO)_3_(**deeb**)**B2**]^+^ could reveal the presence of proteins, as denoted by the luminescent horizontal bands in the respective lane. We did not appreciate a considerable difference by using water (10 min) or DMSO 25% (12 h) to wash the gel, in this case. This result showed that the *fac*-Re(CO)_3_(**deeb**)**B2**]^+^ complex interacts preferentially with proteins instead of the matrix gel. This is a very remarkable point since the matrix gel contains SDS (sodium dodecyl sulfate). The SDS, present in the gel, could interfere with a cationic dye due to its anionic nature, as previously reported (Sundaram et al., 2012). Concerning the free ligand **B2**, which is also luminescent by itself (Carreno et al., 2016a; Berrios et al., 2018; Llancalahuen et al., 2018), we observed an apparent interaction with the gel’s whole matrix, showing intense luminescence even after the wash with water. When DMSO, instead of water, was used to remove unbound **B2**, we could observe some bands of high molecular weight (top of the lane), which generally retain a high amount of dye, in comparison with bands found at the bottom of the lane (low molecular weight). In this case, **B2** was not useful to stain proteins since it does not discriminate appropriately between proteins and the gel. On the other hand, when we tested the rhenium(I) precursor, i.e., *fac*-Re(CO)_3_(**deeb**)Br, we barely could distinguish the bands corresponding to the proteins (Figure 4). All these results together show that the precursor alone virtually is unable to interact with proteins and remain retained, whereas the result obtained with the free ligand **B2** suggests that this compound cannot suitably distinguish between proteins and the gel. Nevertheless, the cationic complex *fac*-[Re(CO)_3_(**deeb**)**B2**]^+^, which is a combination of the precursor and **B2**, showed promising results, allowing for the observation of proteins under the same work conditions.

We speculate that the positive charge of *fac*-[Re(CO)_3_(**deeb**)**B2**]^+^ is contributing to staining proteins. Most protein dyes present an anionic nature, which allows for electrostatic interactions with protonated amino acids (–NH_3_^+^ or –NH^+^–) (i.e*.*, lysine, arginine, or histidine) (Sundaram et al., 2012). Nevertheless, cationic dyes have also been reported as useful to stain proteins, which can be explained by electrostatic interactions with negatively charged residues (e.g., the presence of –COO^−^ in aspartate and glutamate) (Jin and Choi, 2004; Fiorini et al., 2018). We infer that the presence of the **B2** moiety in *fac*-[Re(CO)_3_(**deeb**)**B2**]^+^ could also be contributing to the staining capacity of this complex. It has been reported that hydrophobic interactions, van der Waals forces, and hydrogen bonding can also contribute to the binding of the dye to proteins (Jin and Choi, 2004; Fiorini et al., 2018). In this context, the **B2** moiety could participate in the interaction with proteins due to its capacity to form hydrogen bonds (Carreno et al., 2016a) and perform hydrophobic interactions (e.g*.*, the presence of *tert*-butyls in the benzimidazole ring). In all cases, the *fac*-[Re(CO)_3_(**deeb**)**B2**]^+^ complex forms sufficiently strong interactions with proteins to allow staining that cannot be easily disrupted with either water or even DMSO (the solvent used to dissolve the complex). Another remarkable aspect is the selectivity regarding the interaction preferentially with the proteins and not with the gel matrix, allowing for clear visualization of bands despite the presence of SDS.

In this work, we observed that the *fac*-[Re(CO)_3_(**deeb**)**B2**]^+^ complex could reveal proteins separated in SDS-PAGE with a relatively simple protocol that runs for only 1 h 40 min (including gel washing, staining incubation, and destaining with water), which is a considerable shorter protocol in comparison with other classical techniques, such as CBB or silver stain (3–5 h or even more) (Fiorini et al., 2018). In the case of rhenium(I)-based staining of proteins, including cationic complexes, the reported protocol runs for approximately 14 h (Fiorini et al., 2018), underlining the potential of *fac*-[Re(CO)_3_(**deeb**)**B2**]^+^ as a possible precursor for the development of improved fluorescent stainings, for instance, in combination with another anionic complex to generate a counterion dye (Jin and Choi, 2004). However, more experimentation is needed to improve and further characterize this kind of luminescent dye.

## Conclusion

In this manuscript, we presented the synthesis and characterization of the new *fac*-[Re(CO)_3_(**deeb**)**B2**]^+^ complex. We also assessed photophysical properties, suggesting that **B2** may be a weaker electron-donating group than −Br or may exert a withdrawing effect as an ancillary ligand concerning the Re(I) core. Relativistic studies corroborated these experimental results. Moreover, *fac*-[Re(CO)_3_(**deeb**)**B2**]^+^ also exhibited good features to be used directly as a fluorophore. Finally, we showed the potential of *fac*-[Re(CO)_3_(**deeb**)**B2**]^+^ as a fluorescent protein dye, opening a new window for novel applications of this kind of rhenium(I) tricarbonyl complex.

## Data Availability

The original contributions presented in the study are included in the article/Supplementary Material; further inquiries can be directed to the corresponding authors.
