# Supplementary material for: New Cationic fac-[Re(CO)3(deeb)B2]+ Complex, Where B2 Is a Benzimidazole Derivative, as a Potential New Luminescent Dye for Proteins Separated by SDS-PAGE
Source: Front Chem. 2021 Mar 25;9:647816. doi: 10.3389/fchem.2021.647816 (PMC8027506; doi:10.3389/fchem.2021.647816)
Supplement: Supplementary file 1 [file table1.docx]

**SUPPLEMENTARY MATERIAL**

**New cationic *fac*-[Re(CO)_3_(deeb)B2]^+^ complex, where B2 is a benzimidazole derivative, as a potential new luminescent dye for proteins separated by SDS-PAGE.**

Alexander Carreño,^a^* Manuel Gacitúa,^b^ Eduardo Solis-Céspedes,^c,d^ Dayán Páez-Hernández,^a^ Wesley B. Swords,^e^ Gerald J. Meyer,^e^ Marcelo Preite,^f^ Ivonne Chávez,^g^ Andrés Vega,^h,i^ Juan A. Fuentes^j^*

^a^Center of Applied NanoSciences (CANS), Facultad de Ciencias Exactas, Universidad Andres Bello, República 330, Santiago, Chile.

^b^Facultad de Química y Biología, USACH, Av. L.B. O’Higgins 3363, Santiago, 7254758, Chile.

^c^Escuela de Bioingeniería Médica, Facultad de Medicina, Universidad Católica del Maule.

^d^Laboratorio de Bioinformática y Química Computacional, Facultad de Medicina, Universidad Católica del Maule.

^e^Department of Chemistry, University of North Carolina at Chapel Hill, Chapel Hill, North Carolina 27599-3290, USA

^f^Departamento de Química Orgánica, Facultad de Química y Química y Farmacia, Pontificia Universidad Católica de Chile, Chile

^g^Departamento de Química Inorgánica, Facultad de Química y Química y Farmacia, Pontificia Universidad Católica de Chile, Chile

^h^Departamento de Ciencias Químicas, Facultad de Ciencias Exactas, Universidad Andres Bello, Quillota 980, Viña del Mar, Chile

^i^Centro para el Desarrollo de la Nanociencia y la Nanotecnología, Cedenna, Chile

^j^Laboratorio de Genética y Patogénesis Bacteriana, Facultad de Ciencias de la Vida, Universidad Andrés Bello, República 330, Santiago, Chile.

Corresponding authors: [alexander.carreno@unab.cl](mailto:alexander.carreno@unab.cl); [jfuentes@unab.cl](mailto:jfuentes@unab.cl)

**1. Schemes**

**Scheme S1**. Proton numbering of *fac*-[Re(CO)_3_(**deeb**)**B2**]^+^

**2. Figures**


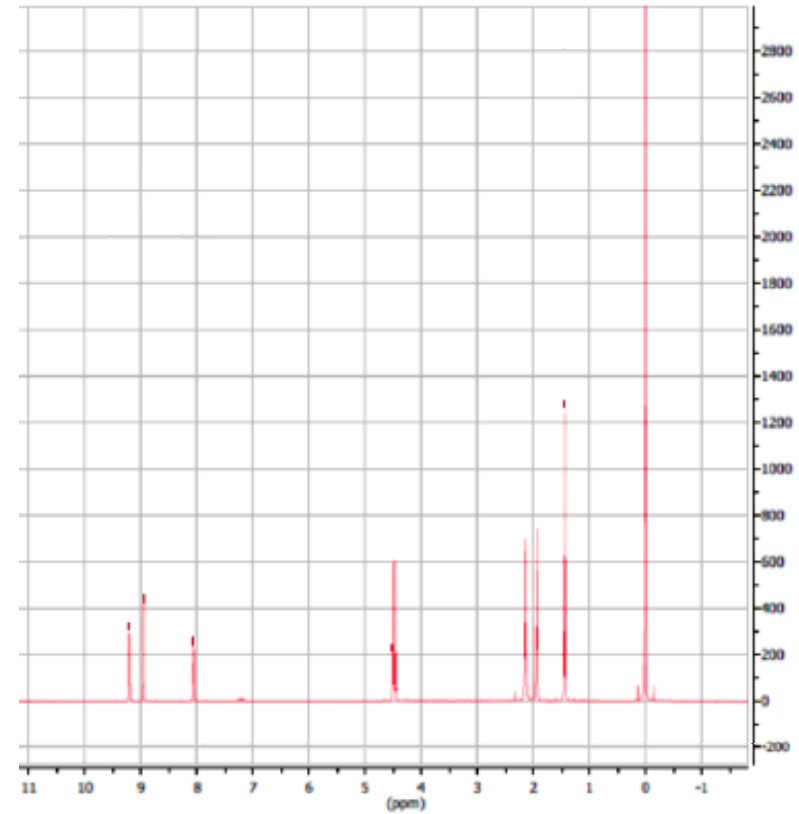


**Figure S1**. ^1^H NMR spectrum of *fac*-Re(CO)_3_(**deeb**)**Br** in acetonitrile-_d3_. [1, 2].


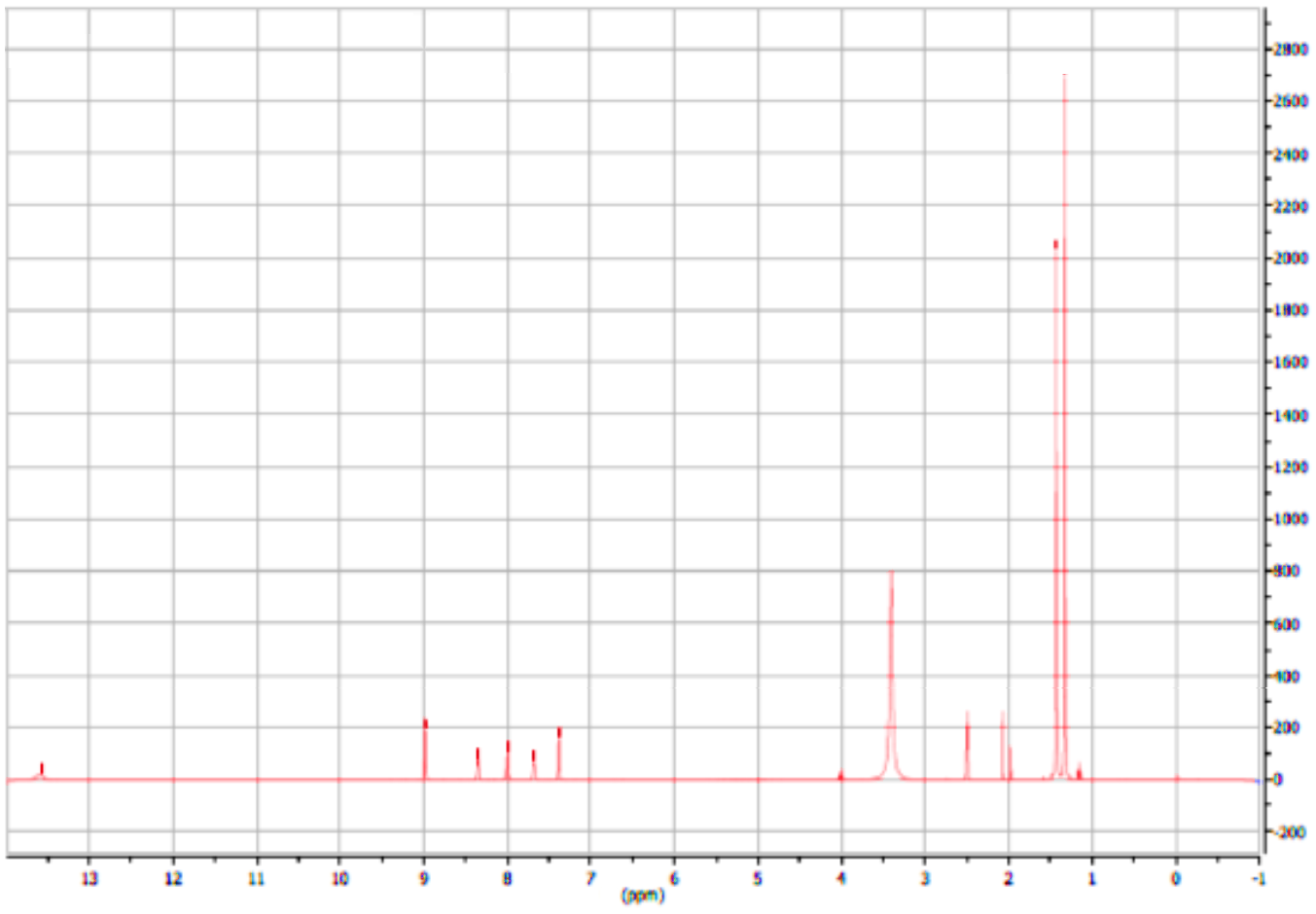


**Figure S2.** ^1^H NMR spectrum of **B2** in DMSO-_d6_ [3, 4].

**Figure S3**. Mass spectrum of *fac*-[Re(CO)_3_(**deeb**)**B2**]^+^.


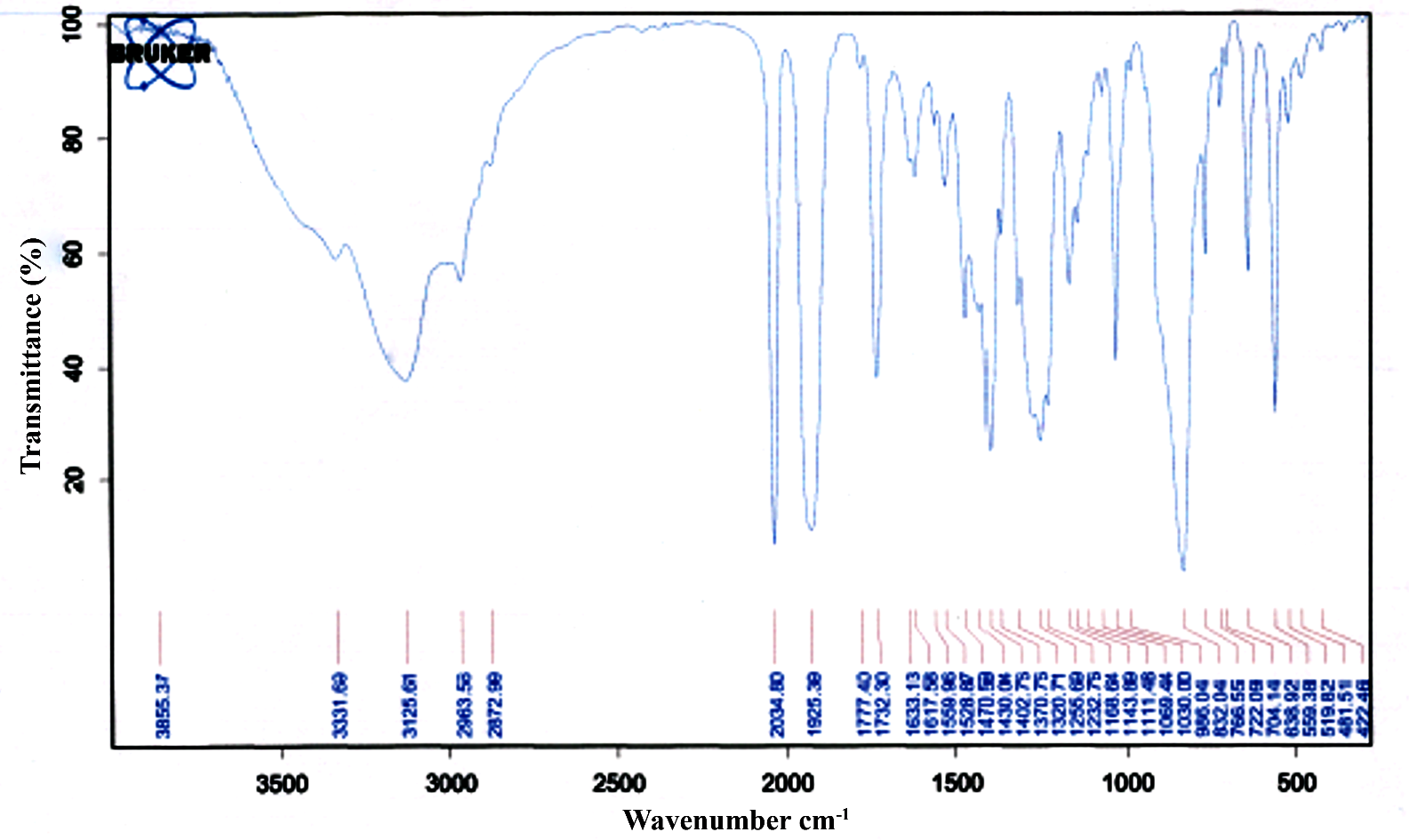


**Figure S4.** FTIR of *fac*-[Re(CO)_3_(**deeb**)**B2**]^+^.

**Figure S5.** ^1^H NMR spectrum of *fac*-[Re(CO)_3_(**deeb**)**B2**]^+^ in acetonitrile-_d3_.

**Figure S6.** Expanded aromatic region of *fac*-[Re(CO)_3_(**deeb**)**B2**]^+^ in acetonitrile-_d3_.

**Figure S7**. D_2_O exchange of *fac*-[Re(CO)_3_(**deeb**)**B2**]^+^ in acetonitrile-_d3_.

**Figure S8**. HHCOSY of *fac*-[Re(CO)_3_(**deeb**)**B2**]^+^ in acetonitrile-_d3_


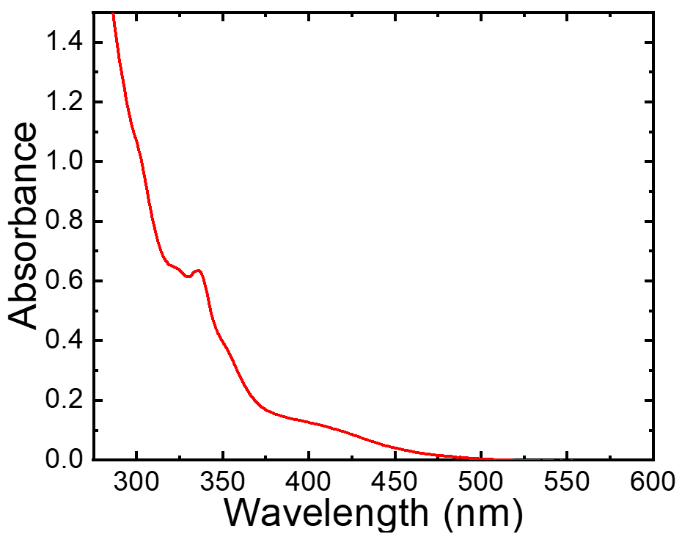


**Figure S9**. Absorption spectra of *fac*-[Re(CO)_3_(**deeb**)**B2**]^+^ in acetonitrile.

**Figure S10.** Working window potential study for *fac*-[Re(CO)_3_(**deeb**)**B2**]^+^. Interphase: Pt|1.0 × 10^-2^ M of compound + 1.0 × 10^-1^ M TBAPF_6_ in anhydrous CH_3_CN.

Working window study is used to determine electrochemical processes peak potential (Ep) and halfwave potential (E_½_). Also, this type of study is used to determine dependency between electrochemical processes found revealing their irreversible (irr) or reversible (rev) character. Working window study for *fac*-[Re(CO)_3_(**deeb**)**B2**]^+^ is presented on **Figure S11**.

**Figure S12.** Cyclic voltammograms for scan-rate study for *fac*-[Re(CO)_3_(**deeb**)**B2**]^+^. Interphase: Pt|1.0 × 10^-2^ M of compound + 1.0 × 10^-1^ M TBAPF_6_ in anhydrous CH_3_CN.

Scan-rate study is used for fast determination if a certain electrochemical process is controlled by mass-transport (diffusion) or not. For each process identified on *fac*-[Re(CO)_3_(**deeb**)**B2**]^+^, cyclic voltammetry runs must be recorded at different scan rates. Then, two plots must be constructed, a “Scan-rate vs current-density peak” and a “(Scan-rate)^½^ vs current density peak” for each reduction and oxidation identified in the system (please see **Table S2**).


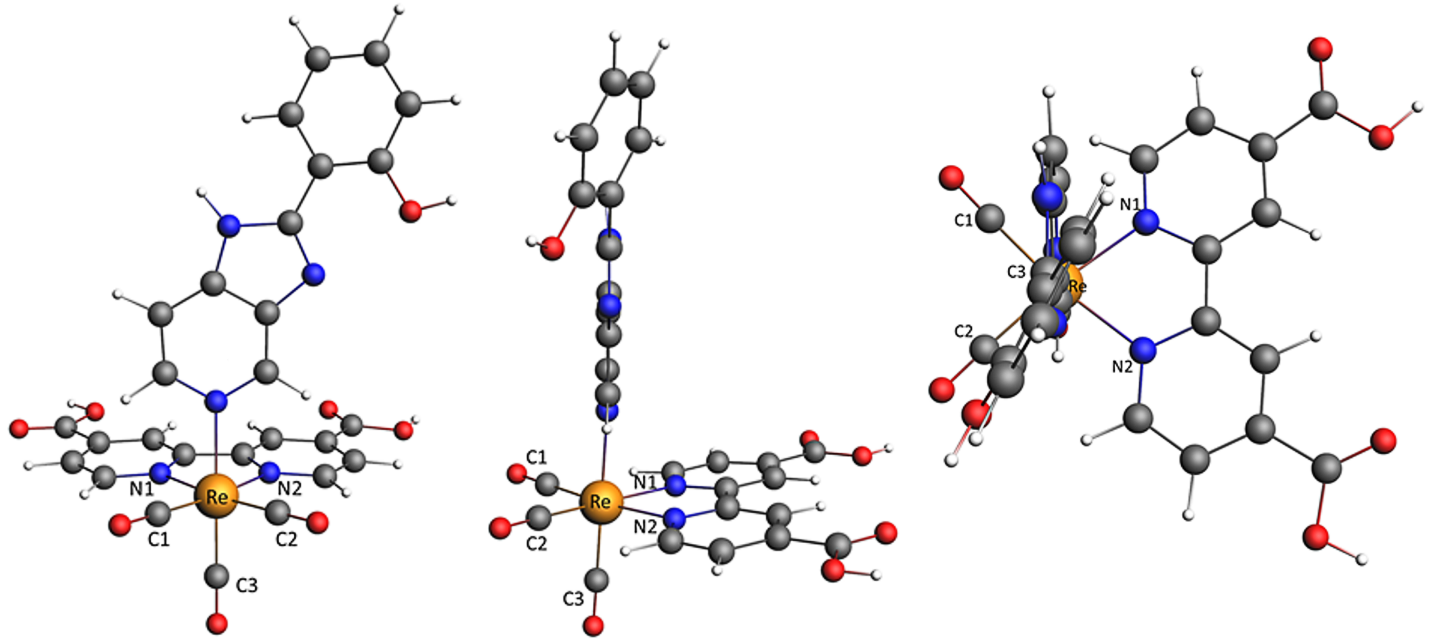


**Figure S13.** Molecular models of *fac*-[Re(CO)_3_(**deeb**)**B2**]^+^ in acetonitrile as solvent. The figure shows the molecule in different views. Atom numbers are depicted.


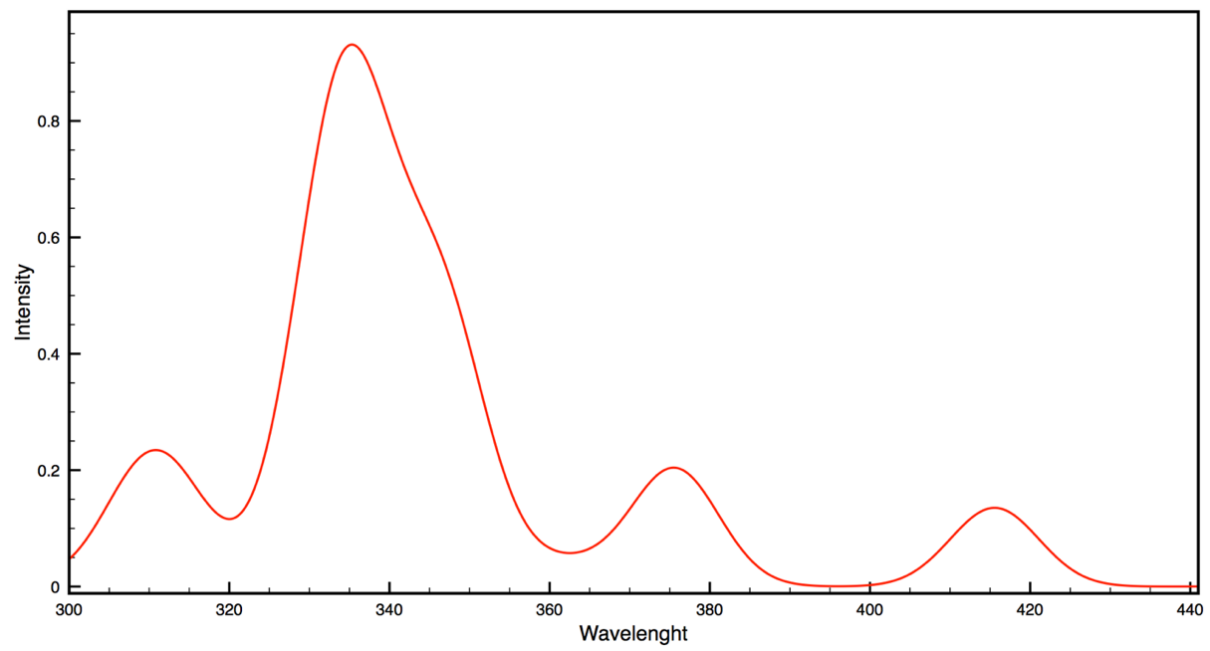


**Figure S14:** Calculated UV-vis absorption spectra of fac-[Re(CO)_3_(**deeb**)**B2**]^+^ in acetonitrile as solvent.

**3. Tables**

**Table S1.** Characteristic constants of *fac*-[Re(CO)_3_(**deeb**)**B2**]^+^

| **Compound** | **Molecular formula** | **M.W. (g mol^-1^)** | **Yield (%)** | **Solid color** |
| --- | --- | --- | --- | --- |
| *fac*-[Re(CO)_3_(**deeb**)**B2**]^+^ | C_39_H_41_N_4_O_8_Re | 893.96 | 70 | orange-brown |

M.W.: Molecular weight

**Table S2.** Scan-rate study results for determining diffusional control of described electrochemical processes at *fac*-[Re(CO)_3_(**deeb**)**B2**]^+^

| Process | Scan-rate vs current-density peak | | | (Scan-rate)^½^ vs current-density peak | | | Diff. control? |
| --- | --- | --- | --- | --- | --- | --- | --- |
|  | m | n | R^2^ | m | n | R^2^ |  |
| $\text{Red}_{\text{(irr)}}^{\text{I}}$ | -5.0 × 10^-6^ | -3.0 × 10^-4^ | 0.900 | -1.0 × 10^-4^ | 1.0 × 10^-4^ | 0.953 | yes |
| $\text{Red}_{\text{(rev)}}^{\text{II}}$ | -5.0 × 10^-6^ | -3.0 × 10^-4^ | 0.930 | -1.0 × 10^-4^ | 7.0 × 10^-5^ | 0.974 | yes |
| $\text{Red}_{\text{(rev)}}^{\text{III}}$ | -3.0 × 10^-6^ | -3.0 × 10^-4^ | 0.940 | -7.0 × 10^-5^ | -4.0 × 10^-5^ | 0.995 | yes |
| $\text{Red}_{\text{(rev)}}^{\text{IV}}$ | -3.0 × 10^-6^ | -5.0 × 10^-4^ | 0.958 | -6.0 × 10^-5^ | -2.0 × 10^-4^ | 0.995 | yes |
| $\text{Ox}_{\text{(irr)}}^{\text{I}}$ | 2.0 × 10^-6^ | 1.0 × 10^-5^ | 0.957 | 3.0 × 10^-5^ | -1.0 × 10^-4^ | 0.990 | yes |
| $\text{Ox}_{\text{(rev)}}^{\text{I}}$ | 3.0 × 10^-6^ | 4.0 × 10^-4^ | 0.969 | 5.0 × 10^-5^ | 2.0 × 10^-4^ | 0.945 | yes |

“m”, “n” and “R^2^” are the slope, intercept and linear regression coefficient, respectively.

**Table S3:** Most important optimized bond distances calculated for fac-[Re(CO)_3_(**deeb**)**B2**]^+^ complex, in acetonitrile as solvent. All distances are in Å.

| **Complex** | **d(Re-C1)** | **d(Re -C2)** | **d(Re -C3)** | **d(Re -B2)** | **d(Re -N1)** | **d(Re -N2)** |
| --- | --- | --- | --- | --- | --- | --- |
| *fac*-[Re(CO)_3_(**deeb**)**B2**]^+^ | 2.004 | 1.973 | 1.998 | 2.193 | 2.172 | 2.129 |

**Table S4:** Carbonyls optimized bond distances calculated for fac-[Re(CO)_3_(**deeb**)**B2**]^+^ complex, in acetonitrile as solvent. All distances are in Å.

| **Complex** | **d(C1-O)** | **d(C2-O)** | **d(C3-O)** |
| --- | --- | --- | --- |
| *fac*-[Re(CO)_3_(**deeb**)**B2**]^+^ | 1.139 | 1.143 | 1.138 |

**Table S5.** Most important optimized bond angles (degrees) calculated for fac-[Re(CO)_3_(**deeb**)**B2**]^+^ complex, in acetonitrile as solvent.

| **N1-Re-C1** | **N2-Re-C2** | **N1-Re-N2** | **C1-Re-C2** | **C1-Re- C3** | **C2-Re- C3** | **B2-Re-C3** | **B2-Re-C1** | **B2-Re-C2** | **B2-Re-N1** | **B2-Re-N2** |
| --- | --- | --- | --- | --- | --- | --- | --- | --- | --- | --- |
| 94.8 | 95.9 | 76.0 | 93.3 | 84.9 | 84.8 | 174.1 | 90.9 | 91.3 | 87.7 | 90.2 |

**References used in the Supplementary Material**

[1] A. Carreño, A.E. Aros, C. Otero, R. Polanco, M. Gacitúa, R. Arratia-Pérez, J.A. Fuentes, Substituted bidentate and ancillary ligands modulate the bioimaging properties of the classical Re(i) tricarbonyl core with yeasts and bacteria, New J. Chem. 41(5) (2017) 2140-2147.

[2] A. Carreno, M. Gacitua, E. Schott, X. Zarate, J.M. Manriquez, M. Preite, S. Ladeira, A. Castel, N. Pizarro, A. Vega, I. Chavez, R. Arratia-Perez, Experimental and theoretical studies of the ancillary ligand (E)-2-((3-amino-pyridin-4-ylimino)-methyl)-4,6-di-tert-butylphenol in the rhenium(I) core, New Journal of Chemistry 39(7) (2015) 5725-5734.

[3] A. Carreno, M. Gacitua, J.A. Fuentes, D. Paez-Hernandez, C. Araneda, I. Chavez, M. Soto-Arriaza, J.M. Manriquez, R. Polanco, G.C. Mora, C. Otero, W.B. Swords, R. Arratia-Perez, Theoretical and experimental characterization of a novel pyridine benzimidazole: suitability for fluorescence staining in cells and antimicrobial properties, New Journal of Chemistry 40(3) (2016) 2362-2375.

[4] F.M. Llancalahuen, J.A. Fuentes, A. Carreno, C. Zuniga, D. Paez-Hernandez, M. Gacitua, R. Polanco, M.D. Preite, R. Arratia-Perez, C. Otero, New Properties of a Bioinspired Pyridine Benzimidazole Compound as a Novel Differential Staining Agent for Endoplasmic Reticulum and Golgi Apparatus in Fluorescence Live Cell Imaging, Frontiers in Chemistry 6 (2018).
